# Familiarizing with science editors' associations

**DOI:** 10.3325/cmj.2011.52.735

**Published:** 2011-12

**Authors:** Armen Yuri Gasparyan

## Abstract

The number of science editors' associations is growing to resolve a variety of professional problems. The main objective of the associations is to educate their members by facilitating dissemination of information, publishing scholarly journals, books, networking of experts, and regular meetings. There are many science editing associations – general and specialized, traditional, and new. The article presents activities of some of these associations, which include upholding standards of science writing, editing, indexing, research reporting, peer review, editorial independence, and other editorial policies.

The number of scholarly journals constantly increases in order to meet the ever changing demands of academic institutions, learned societies, and groups of professionals in different fields of science. It now reaches a level above 200 000 (1). The main objectives of journals struggling to become influential in their field are to disseminate information, facilitate science communication among specialists (2), and get indexed in prestigious indexing databases by publishing high-quality and citable items (3). These objectives are interconnected, and ideally should be based on universal principles of integrity and honesty (4,5). Integrity derives from the Latin word *integer*, meaning complete, perfect, whereas honesty – from the Sanskrit word *hunar* (good men), or Latin word *honos* (an individual abiding to the code of conduct accepted by society at large).

In modern-day science editing, both integrity and honesty are defined by professional societies accumulating wisdom, experience, and skills, and supporting individual researchers and editors in their efforts to make their field of science more advanced.

The role of science editors' societies is evolving to solve the numerous problems of efficient writing, editing, and publishing. The exponential growth of scholarly publications and the digitalization of the whole publishing industry dictate the necessity of launching new organizations or expanding the functions of old ones. As such, it is not surprising that there is a variety of international, regional, and local associations, each concerned with unique and sometimes overlapping sets of writing and editing challenges (eg, unbiased reporting of research data, peer review, authorship, and plagiarism). Some of the international associations are presented in Table 1. Knowledge of the functions of these associations, offered educational resources, and other benefits of membership is central to the success of running scholarly journals (6). Familiarizing with the associations and their educational resources will help authors, reviewers, and editors to advance their skills and to achieve better results.

**Table 1:** Activities of science editors' professional associations

Title and Web site	Publications	Other resources	Events	Discussion lists
American Copy Editors Society (ACES) www.copydesk.org	-	Forums	Annual conferences	+
American Medical Writers Association (AMWA) www.amwa.org	*AMWA Journal* *AMWA Update* e-newsletter	Essays for biomedical communicators Self-study workshops on ethics, grammar, statistics, etc. Webinars	Annual conferences	+
American Society of Healthcare Publication Editors (ASHPE) www.ashpe.org	*Outlook* newsletter	ASPHE Code of ethics and preferred editorial practices	-	-
Association of Learned and Professional Society Publishers (ALPSP) www.alpsp.org	*Learned Publishing* journal E-newsletter	Online courses Webinars	Annual conferences Seminars	+
Board of Editors in the Life Sciences (BELS) www.bels.org	*-*	Editor in the Life Sciences (ELS) certification exam Diplomate (ELS [D]) and honor (ELS [H]) programs	-	-
Committee on Publication Ethics (COPE) http://publicationethics.org	*Ethical Editing* newsletter	COPE's code of conduct flowcharts Guidelines Cases on misconduct	Annual seminars in the UK and the USA	+
Council of Editors of Learned Journals (CELJ) www.celj.org	*-*	Guidelines	Modern Language Association's annual meetings	+
Council of Science Editors (CSE) www.councilscienceeditors.org	*Science Editor* journal	CSE's white paper on integrity in journal publications Guidelines Webinars	Annual meetings	+
European Association of Science Editors (EASE) www.ease.org.uk	*European Science Editing* journal	Handbook for editors Guidelines for authors and translators Blog	Triennial congresses	+
European Medical Writers Association (EMWA) www.emwa.org	*The Write Stuff* journal	Blog	Biannual conferences Workshops	+
Enhancing the QUALity and Transparency Of health Research network (EQUATOR) www.equator-network.org	-	Webinars Digital library on health research reporting	Seminars and lectures on reporting of research results (CONSORT, STARD, PRISMA and other statements)	+
International Academy of Nursing Editors (INANE) www.nursingeditors-inane.org	*Nurse Author & Editor* newsletter	Blogs	Annual conferences	+
International Association of Scientific, Technical and Medical Publishers (STM) www.stm-assoc.org	*STM news* newsletter	Guidelines Video films	Seminars Training courses	+
International Committee of Medical Journal Editors (ICMJE) www.icmje.org	-	Uniform requirements for manuscripts submitted to biomedical journals Uniform disclosure form for potential conflicts of interest Updates on clinical trial registration	Annual meetings	-
International Society of Managing and Technical Editors (ISMTE) www.ismte.org	*ISMTE newsletter* *Best Practices for the Editorial Office News* newsletter	ISTME's publishing terms glossary Video films on high-quality figures	Biannual conferences	-
International Society for Medical Publication Professionals (ISMPP) www.ismpp.org	Code of ethics Positions statements	Certified Medical Publication Professional exam and certification valid for 5 y	Annual meetings Workshops	+
Mediterranean Editors & Translators www.metmeetings.org	-	News Webinars Tutorials	Annual meetings Workshops	+
National Association of Science Writers (NASW) www.nasw.org	*Science Writers* magazine	NASW Code of ethics for journalists	Annual conferences and workshops	+
Office of Research Integrity (ORI) http://ori.dhhs.gov	*Office of Research Integrity* newsletter	Guidelines, handbooks, reports on misconduct, video films	Conferences Seminars	+
Society for Editors and Proofreaders (SfEP) www.sfep.org.uk	*Editing Matters* magazine	SfEPWiki repository of the Society's experience StEP's code of practice Accreditation in proofreading	Annual conferences Proofreading courses	+
Society for Technical Communication (STC) www.stc.org	*Technical Communication* journal *Intercom* magazine News & Notes e-newsletter	Online conferences and seminars STC's Notebook blog	Annual conferences Certificate courses in technical communication	+
World Association of Medical Editors (WAME) www.wame.org	-	Conflict of Interest in Peer-Reviewed Medical Journals The Relationship Between Journal Editors-in-Chief and Owners Other policy and publication ethics statements Syllabus for Editors	-	+
World Federation of Science Journalists (WFSJ) www.wfsj.org	-	Online course in science journalism Science Journalism Blog Books	Biennial conferences	+

Though science editing is a field of science itself, it is still poorly represented in the academic community, and subsequently editors' credentials are not uniformly accepted. Throughout the world, there are only a few accredited and internationally recognized degree and non-degree programs dealing with the core content of research methodology and editing (7,8). The knowledge base and curricula of these courses predominantly stem from information presented and discussed within learned societies. Likewise, there are a few prestigious short courses gathering scientific and technical editors and run by distinguished members of the learned societies. A good example is the 2-day course “How to be a successful journal editor” regularly arranged by PSP consulting in Oxford, UK and elsewhere in Europe (9). Apart from these, editors around the world are offered different educational meetings, conferences, and congresses as part of their membership in professional associations.

European Association of Science Editors (EASE) is one of the leading organizations fostering editors' continuous education. It is a scientific member of the International Union of Biological Sciences, the International Union of Geological Sciences (IUGS), the International Council of Science Unions (ICSU), and is in formal associate relations with UNESCO. Perhaps the main indispensable benefit offered to members of EASE from Europe and rest of the world is the triennial congress. Another essential source of information is the EASE guideline for authors and translators available on the EASE Web site (10). It is also printed in the 3rd issue of *Acta Informatica Medica* for 2011. Last, but not least important, are the EASE handbook for science editors and *European Science Editing* quarterly journal. The journal is widely indexed by a number of online catalogs, databases, and digital libraries to serve broad spectrum of interests of editors from different professional backgrounds. It covers a variety of issues in science communication, publishing ethics, scientometrics, and informatics (11).

Council of Science Editors (CSE; formerly Council of Biology Editors [CBE]) is the US-based association promoting excellence in editorial policies, peer review, and publication ethics. Among a variety of educational sources (12), CSE offers *Science Editing*, a quarterly journal, and the regularly updated “White Paper on Promoting Integrity in Scientific Journal Publications,” the set of guidelines on editorial policy adopted by editors and publishers of influential biomedical and other journals such as *Science, Cell, The New England Journal of Medicine, Proceedings of the National Academy of Sciences of the United States of America,* and *Annals of Internal Medicine*. The document comprehensively covers functions of editors and peer reviewers, authorship criteria, conflicts of interest, and scientific misconduct. It is primarily of interest to biomedical science editors.

High standards of medical communication are advocated by American Medical Writers Association (AMWA) and European Medical Writers Association (EMWA). Both organizations publish quarterly journals, arrange annual meetings, and a series of workshops. On the basis of its workshops, AMWA published two volumes of “Essays for biomedical communicators” presenting effective writing and language editing tips for authors and editors. Also, AMWA introduced a series of self-study workshops or educational modules on ethics, medical terminology, basics of English grammar and punctuation, structuring sentences, and biostatistics. The modules presented as CD-ROMs are invaluable educational tools for medical communicators.

International Committee of Medical Journal Editors (ICMJE) is a working group of several general medical journals, also known as the Vancouver group. Its main document, “Uniform Requirements for Manuscripts Submitted to Biomedical Journals,” is a primary source of information on publishing ethics, copyright issues, manuscript structuring, and formatting for authors, peer reviewers, and editors of biomedical journals (13). The majority of the journals follow these recommendations and build up their editorial policy accordingly. ICMJE also proposed the “Uniform Disclosure Form for Potential Conflicts of Interest” (14).

Committee on Publication Ethics (COPE) is the UK-based group of editors. It has worldwide representation, particularly among editors of medical journals, and the membership of journals published by Elsevier, Springer, Informa Healthcare, DovePress, and other major publishers. COPE published 17 flowcharts for editors on how to handle disputes over changes in authorship, undisclosed conflict of interest, suspected plagiarism and duplicate publication, data fabrication, and complaints against editors (15). It also published a set of guidelines for new editors, code of conduct for editors and publishers, and guidelines on article retraction. All of which, and others, are available on their Web site. Importantly, COPE's resources for editors are increasingly used by members and non-members, declaring adherence to best practice guidelines in their instructions for authors.

Medical editors may also benefit from resources offered by the Enhancing the QUALity and Transparency Of health Research (EQUATOR) Network. EQUATOR's staff is formed by experts from the Centre for Statistics in Medicine, Oxford, UK. Its main goal is to promote transparent and accurate reporting of research data by educating authors, peer reviewers, and editors. The digital library of the Network contains numerous publications on science writing and links to reporting guidelines presented in the form of checklists and flow diagrams: CONSORT (CONsolidated Standards of Reporting Trials), STARD (STAndards for the Reporting of Diagnostic accuracy studies), PRISMA (Preferred Reporting Items for Systematic Reviews and Meta-Analyses) statements, STROBE (STrengthening the Reporting of OBservational studies in Epidemiology) checklist for cohort, case-control, and cross-sectional studies, etc. These statements are endorsed by learned societies such as CSE and the World Association of Medical Editors (WAME) and incorporated as a part of editorial policies in numerous biomedical journals.

World Association of Medical Editors (WAME) is the leading organization upholding standards of biomedical editing worldwide. Its membership is free and open to all those with experience in scientific and technical editing in big or small journals. Perhaps the main benefit of membership is the access to Listserve discussions, offering solutions to a variety of problems encountered by editors in their editorial practice. Also, WAME has archived its Listserve discussions and issued policy statements on editorial independence, conflict of interest, impact factors, authorship, etc. Novice editors may advance their skills by adhering to the guidelines presented in Syllabus for Prospective and Newly Appointed Editors. Adherence to WAME's statements and the use of other related resources by members is strongly encouraged, and may be helpful for increasing the quality and widening indexing of their journals.

To meet specific needs of medical editors from different countries and regions, several national and regional associations were launched in the past decade. The Eastern Mediterranean Association of Medical Editors (EMAME) and the Forum for African Medical Editors (FAME) are good examples of the regional organizations with specific scope of interests and goals. Improving medical writing skills, avoiding plagiarism, and arranging fair peer review are top priorities for both EMAME and FAME. Both regional associations regularly arrange meetings and publish guidelines addressing specific issues of research culture and integrity in science scholarship (16).

In the era of digitalization and changing requirements toward online and print publication, science editors are under pressure to keep up with the pace of changes (3,17).

Learning new information technologies, switching to electronic editorial management, improving publishing standards, and meeting indexing criteria of citation-tracking databases such as Science Citation Index-Expanded (SCI-E) and Social Science Citation Index (SSCI) is becoming a matter of survival and further development for most scholarly journals. Available educational resources of most science editing organizations scarcely cover these issues. The Association of Learned and Professional Society Publisher**s** (ALPSP) is one of the few organizations regularly providing updates and advice on how to meet increasingly demanding indexing criteria of prestigious databases. In this regard, *Learned Publishing*, the quarterly journal of the Association, can be viewed as a must-read periodical for editors from different backgrounds.

Along with many helpful resources of the learned societies, some of which are presented in Table 1, networking of specialists remains the most feasible way of keeping up-to-date and resolving daily problems. Social networking sites such as Facebook, LinkedIn, and Twitter partly fulfill the function of distributing professional information by editors of most associations. Furthermore, Listserves, forums, blogs, online workshops, and other options of digital networking are becoming usual attributes of well-organized societies of editors. At the same time, traditional conferences, seminars, and personal meetings hold strong positions as a culmination of networking. Importantly, these meetings are not limited to the editors' societies. Other professional associations such as the European League Against Rheumatism (EULAR) arrange editorial sessions as a part of the scientific program of their annual congresses. In fact, the EULAR 2011 congress in London included a session on science writing and peer review for a non-expert audience, run by editors of leading rheumatology journals, *Arthritis and Rheumatism, Annals of the Rheumatic Diseases,* and *Rheumatology*.

In conclusion, current flow of scientific information and constant growth of scholarly publications require a more systematic approach toward the education of science editors. In this regard, learned societies play the central role by facilitating distribution of information and networking, developing guidelines and policy statements, advocating the editors' interests, conducting research, and publishing periodical literature. To unite specialists with common professional, regional, social, educational, and other needs, and to resolve a variety of the ever changing problems, new associations and networks are forming, some of them on the basis of old ones. Here we are reminded of Albert Einstein's famous quote: “Life is like riding a bicycle. To keep your balance you must keep moving.”
